# Nonarteritic anterior ischemic optic neuropathy is associated with cerebral small vessel disease

**DOI:** 10.1371/journal.pone.0225322

**Published:** 2019-11-14

**Authors:** Min Seok Kim, Han Yeong Jeong, Kwan Hyuk Cho, Seung Won Oh, Seong Jun Byun, Se Joon Woo, Hee Kyung Yang, Jeong Min Hwang, Kyu Hyung Park, Chi Kyung Kim, Sang Jun Park

**Affiliations:** 1 Department of Ophthalmology, Seoul National University College of Medicine, Seoul National University Bundang Hospital, Seongnam, South Korea; 2 Department of Neurology, Seoul National University Hospital, Seoul, South Korea; 3 Retina Center, Moon's Eye Clinic, Suwon, South Korea; 4 Department of Family Medicine, Healthcare System Gangnam Center, Seoul National University Hospital, Seoul, South Korea; 5 School of Pharmacy, Sungkyunkwan University, Suwon, Republic of Korea; 6 Department of Neurology, Korea University Guro Hospital and College of Medicine, Korea University, Seoul, South Korea; Instituto Mexicano del Seguro Social (IMSS) HGZ 2, MEXICO

## Abstract

We investigated the presence of cerebral small vessel disease (SVD) in patients with nonarteritic anterior ischemic optic neuropathy (NAION) compared to control subjects without NAION to identify the association between NAION and cerebral SVD. We retrospectively reviewed the cases of 63 patients with NAION and 2749 control subjects without any neurologic and ocular diseases including NAION who underwent careful medical interviews, ophthalmic examinations, and magnetic resonance imaging (MRI) studies of the brain. We assessed and compared the degree of cerebral SVD on the MRIs. The patients with NAION presented with cerebral SVD more frequently than controls (68% versus 37%, respectively, p<0.001), which was also observed after adjusting for age, sex, comorbid conditions including hypertension, diabetes, and dyslipidemia, and smoking using the standardized mortality ratio (68% vs. 37%, p<0.001). A multivariate logistic regression analysis showed that the odds of cerebral SVD were 4.86 (95% CI, 2.10 to 11.24, p<0.001) times higher in patients with NAION than in the controls. We found that there was an association between cerebral SVD and NAION even after adjusting for age, sex, and medical histories. Clinicians should consider brain MRI scans in patients with NAION to prevent neurological impairment after cerebral SVD.

## Introduction

Cerebral small vessel disease (SVD) refers to a group of pathologic processes that affect the small arteries and arterioles of the brain,[1] and is related to future physical disabilities secondary to stroke or parkinsonism, and future cognitive decline secondary to dementia.[2, 3] While it is difficult to visualize cerebral SVD pathologies *in vivo*, the diagnosis of cerebral SVD is readily established using brain magnetic resonance imaging (MRI) findings including white matter hyperintensities (WMH), cerebral microbleeds (CMB), and silent lacunar infarcts.[4]

Nonarteritic anterior ischemic optic neuropathy (NAION) is the second most common optic neuropathy in elderly patients after glaucoma. It is characterized by sudden and painless visual loss.[5, 6] NAION is a multifactorial disease that causes hypoperfusion of the short posterior ciliary arteries (SPCAs).[7] A retrobulbar hemodynamic study confirmed considerable decreases in blood flow velocities through SPCAs in patients with NAION,[8] and another study using fluorescein angiography reported that blood flow impairments were observed, not in the SPCAs themselves, but in the para-optic branches of the SPCAs.[9] Therefore, NAION could be considered to involve small arteries and arterioles. Historically, studies of the association between NAION and stroke have focused mainly on thromboembolic mechanisms, which are generally related to large vessel disease. Virtually, nothing is known about the association between SVD and NAION. Therefore, in this study, we thoroughly assessed the distribution and degree of cerebral SVD in patients with and without NAION to investigate the association between NAION and cerebral SVD, and to provide evidence to support the hypothesis that the two diseases share a common pathophysiology.

## Materials and methods

This study was approved by the institutional review boards of Seoul National University Bundang Hospital (B-1506-304-114) and Seoul National University Hospital (SNUH) Healthcare System Gangnam Center (J-1609-065-791), and was carried out in accordance with the tenets of the Declaration of Helsinki. Informed consent was waived due to the retrospective nature of the study.

**Fig 1:**
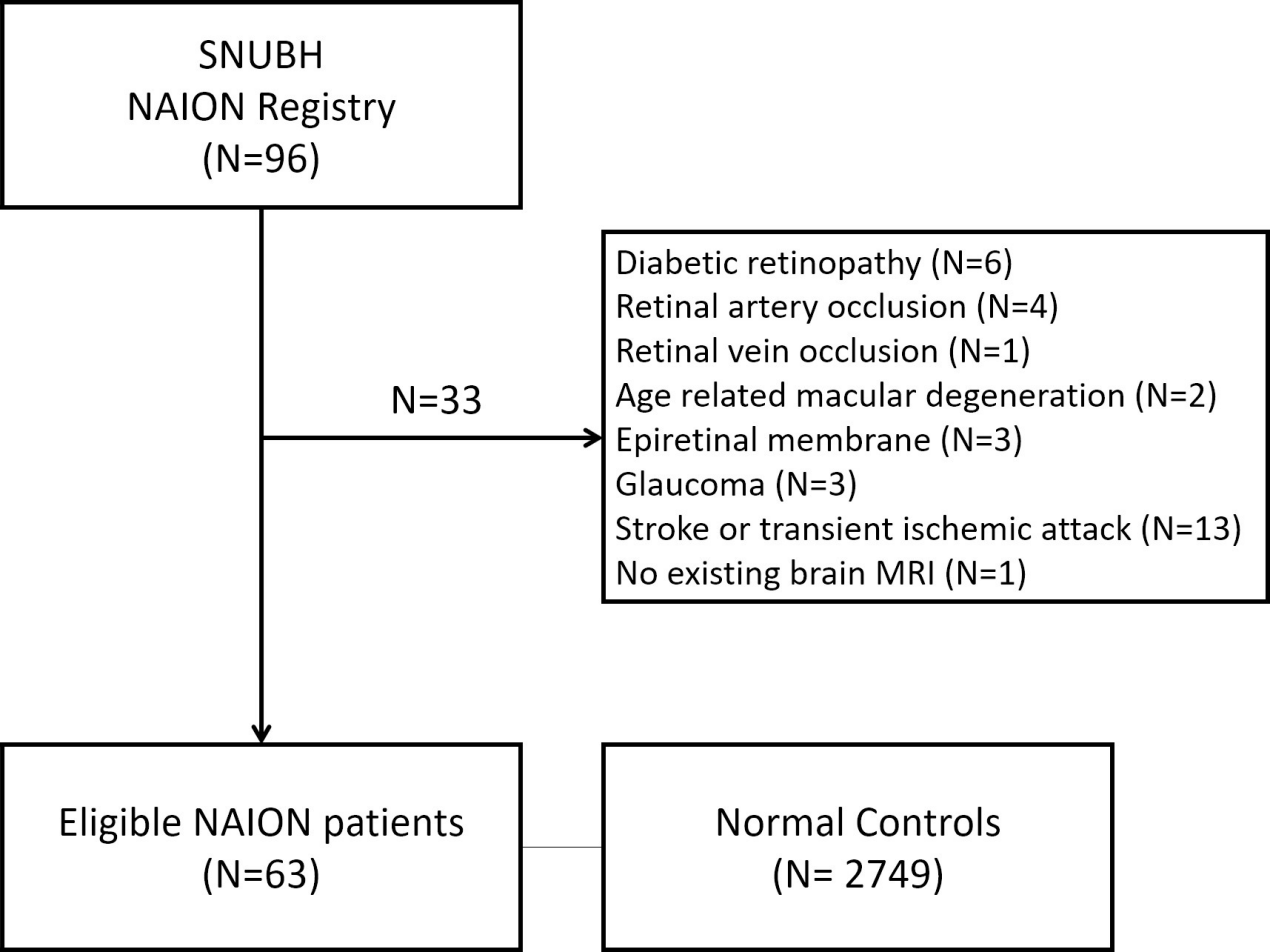
Flow diagram showing the selection for the enrolled patients with nonarteritic anterior ischemic optic neuropathy. The controls included individuals who had attended Seoul National University Hospital Healthcare System, Gangnam Center for routine health screening. SNUBH: Seoul National University Bundang Hospital; NAION: nonarteritic anterior ischemic optic neuropathy; MRI: magnetic resonance imaging.

We retrospectively reviewed the medical records of patients with NAION who consecutively visited Seoul National University Bundang Hospital from June 2003 and May 2016. Two neuro-ophthalmologists (HKY and JMH) confirmed the diagnosis of NAION with these inclusion criteria: sudden, painless loss of visual acuity without history of glaucoma or retinal diseases, optic disc edema with or without superficial hemorrhages at the optic disc border and adjacent retina on fundus ophthalmoscopy, or visual field defects consistent with NAION. Of 96 patients diagnosed with NAION, we excluded 6 patients with diabetic retinopathy, 4 with retinal artery occlusion, 1 with retinal vein occlusion, 2 with age-related macular degeneration, 3 with epiretinal membrane, 3 with glaucoma, 13 with any neurological diseases (e.g. stroke or transient ischemic attack) and 1 patient without a brain MRI scan between 1 year before and after the NAION diagnosis. Therefore, we included a total of 63 patients with NAION in our analysis (Fig 1).

### Demographics, medical histories, and ophthalmic evaluations

All patients underwent slit-lamp bio-microscopy, indirect ophthalmoscopy, and fundus photography (Vx-10; Kowa Optimed, Tokyo, Japan; Optos PLC, Dunfermline, Scotland, UK) at the initial visit. We obtained demographic information and information on hypertension, diabetes mellitus, dyslipidemia, and smoking status through a detailed review of all the patients’ medical charts to assess their vascular risk factors.

### Controls

We identified the control group for this study by retrospectively reviewing the charts of subjects who underwent routine health checkups at the SNUH Healthcare System, Gangnam Center between October 2003 and December 2004.[10, 11] Controls were enrolled from the general population of the same region with the patient group in the Southeast part of Seoul Metropolitan area. They had no neurological diseases (e.g. stroke and transient ischemic attack) and any abnormal findings in the fundus examination including optic disc. We consecutively reviewed the medical records of each subject that included comprehensive interviews and physical examinations, and laboratory results. We included a total of 2749 healthy individuals in the control group, and recorded their demographic information and current disease information including hypertension, diabetes mellitus, dyslipidemia, and smoking status.

### Evaluation of brain MRI scans

**Fig 2:**
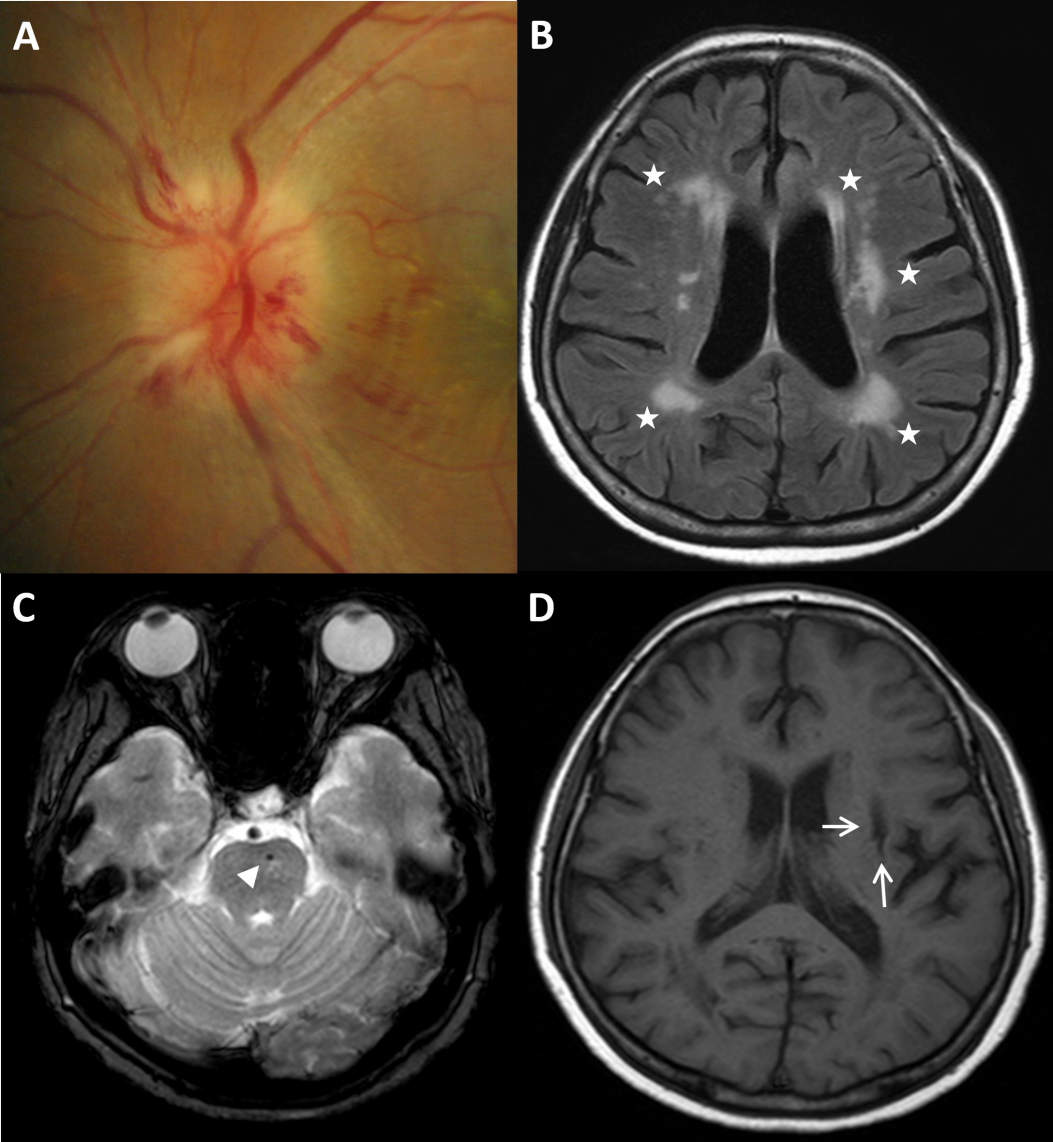
Cerebral small vessel disease in a patient with NAION. (A) A case of NAION with hyperemic disc, segmental swelling, and disc hemorrhage in a 62-year-old woman. (B) White matter hyperintensities (Fazekas grade 3) on brain MRI (FLAIR image: white asterisks). (C) Cerebral microbleeds on T2-weighted brain MRI (white arrowhead) (D) silent lacunar infarct on T1-weighted MRI (white arrow) corresponding to cerebral small vessel disease. NAION: nonarteritic anterior ischemic optic neuropathy; MRI: magnetic resonance imaging; FLAIR: fluid attenuated inversion recovery.

We evaluated the cerebrovascular status of the NAION and control groups using a 1.5 or 3.0 Tesla MRI scanner (Interaor Achieva; Philips, Best, The Netherlands; Signa; GE, Milwaukee, WI, USA) and 1.5 Tesla Chorus MRI scanner (ISOL Technology, Inc., Kyungki-Do, Republic of Korea). Detailed descriptions of the MRI protocols and SVD findings on the brain MRIs were published in our previous studies.[11, 12] Briefly, MRI protocols included T1-weighted, T2-weighted, T2* gradient-recalled echo (GRE), diffusion-weighted, and fluid-attenuated inversion recovery (FLAIR) images. WMH were defined as areas of bright, high-signal intensities noted on T2-weighted images and graded by Fazekas score.[13] We defined CMB as black round lesions with a blooming artifact on T2* gradient images with diameters within 5 mm.[14, 15] Silent lacunar infarction was defined by a hyperintense signal on T2-weighted or FLAIR images and a hypointense signal on T1-weighted images, often surrounded by a hyperintense signal rim on FLAIR images.[16] Cerebral SVD was defined by one or more of the following: grade 1 or greater WMH, CMB, or silent lacunar infarcts (Fig 2). Two independent trained stroke neurologists (CKK and HYJ) who were blinded to the clinical information assessed the degree of WMH, CMB, and silent lacunar infarcts on the brain MRIs. When discrepancies arose, the two observers discussed their evaluations and came to an agreement.

### Statistical analysis

To reduce the impact of demographics, underlying medical conditions, and smoking status, we performed a rigorous adjustment using the propensity score (PS)-based standardized mortality/morbidity ratio (SMR) weight technique. Other PS techniques such as matching and stratification were not used because of the selection bias caused by inevitably discarded data. The SMR weights standardized the control population to the NAION population by creating a pseudo-population of the controls, which had the same covariate distribution as the patients with NAION. Table 1 provides the distribution of demographics, past medical histories, and smoking statuses before and after the SMR-based standardization. We estimated the distribution of SVDs (WMH, CMB, and silent lacunar infarcts) in the SMR-based standardized control population and performed a logistic regression analysis to delineate the association between NAION and the presence of SVDs (WMH, CMB, and silent lacunar infarcts) according to SMR weights. We also performed another standardization method using inverse probability of treatment weights (IPTW) before applying the SMR weight-based method; however, we could not balance the covariates between the NAION patients and the controls using the IPTW-based standardization. All reported p values are 2-sided, and values of p < 0.05 were considered to indicate statistical significance. We used SAS software version 9.3 (SAS Inc., Cary, NC, USA) and R programming version 3.1.0 (The R Foundation for Statistical Computing, Vienna, Austria, http://www.R-project.org) for all analyses.

**Table 1:** Demographics and brain MRI findings of patients with NAION and controls before and after adjusting with SMR.

Characteristics	Unadjusted Data			Adjusted by SMR		
	NAION n = 63	Control n = 2749	*p*-value	NAION	Control	*p*-value
Age, yr	59.1±11.3	61.5±10.7	0.1	59.1±11.3	59.1±1.58	0.941
Sex, men, n (%)	30 (48)	1602 (58)	0.117	(48)	(48)	0.985
**Past medical history, n (%)**						
Hypertension	28 (45)	936 (34)	0.113	(44)	(44)	0.981
Diabetes mellitus	17 (27)	405 (15)	0.012*	(27)	(27)	0.999
Dyslipidemia	11 (18)	346 (13)	0.338	(17)	(17)	0.912
Smoking	12 (19)	1890 (69)	<0.001*	(19)	(19)	0.993
**Cerebral SVD n(%)**	43 (68)	1024 (37)	<0.001*	(68)	(37)	<0.001*
WMH	38 (60)	861 (31)	<0.001*	(60)	(31)	<0.001*
Grade 0	25 (40)	1888 (69)		(40)	(69)	
Grade 1	28 (44)	717 (26)		(44)	(27)	
Grade 2	6 (10)	117 (4)		(10)	(4)	
Grade 3	4 (6)	27 (1)		(6)	(0)	
CMB	8 (13)	191 (7)	0.083	(13)	(6)	0.198
Silent lacunar infarct	11 (17)	281 (10)	0.098	(17)	(10)	0.205

Continuous variables are reported as the mean ± standard deviation.

* p< 0.05

MRI: magnetic resonance imaging; NAION: nonarteritic anterior ischemic optic neuropathy; SMR: standardized mortality ratio; SVD: small vessel disease; WMH: white matter hyperintensities; CMB: cerebral microbleeds

## Results

### Unadjusted data

We included a total of 63 patients (30 men, 48%) with NAION, and 2749 control participants without NAION (1602 men, 58%). The average ages were 59.1 (range, 36–82 years) for the NAION group at the time of the initial diagnosis, and 61.5 (range, 20–96 years) for the control group (p = 0.1). We compared the demographics, past medical histories, and brain MRI findings that were significant for SVD between the NAION and control groups. The prevalence of diabetes mellitus was significantly higher in the NAION group than in the control group, but there was no significant difference in hypertension and dyslipidemia. Controls were more likely to smoke than the patients with NAION. In addition, patients with NAION showed a significantly higher prevalence of cerebral SVD than controls (Table 1). The multivariate logistic regression analysis showed that the odds ratio (OR) of cerebral SVD was 4.81 times higher among the patients with NAION than the controls (95% confidence interval [CI], 2.68–8.66) (Table 2).

**Table 2:** Multivariate logistic regression results for small vessel disease before SMR adjustment.

Variables	All cerebral SVD		WMH		CMB		Silent lacunar infarct	
	Odds ratio (95% CI)	p-value	Odds ratio (95% CI)	p-value	Odds ratio (95% CI)	p-value	Odds ratio (95% CI)	p-value
NAION	4.81 (2.68–8.66)	<0.001	4.50 (2.53–8.01)	<0.001	2.23 (1.05–5.17)	0.04	2.22 (1.07–4.61)	0.033
Age (yr)	1.07 (1.06–1.08)	<0.001	1.08 (1.07–1.09)	<0.001	1.03 (1.02–1.05)	<0.001	1.07 (1.05–1.09)	<0.001
Sex (men)	0.80 (0.66–0.96)	0.016	0.73 (0.60–0.88)	0.001	1.26 (0.918–1.74)	0.168	1.07 (0.82–1.40)	0.623
Hypertension	1.89 (1.58–2.25)	<0.001	1.91 (1.59–2.29)	<0.001	1.78 (1.31–2.42)	<0.001	2.15 (1.65–2.79)	<0.001
Diabetes	1.24 (0.99–1.56)	0.062	1.25 (0.99–1.58)	0.057	0.95 (0.64–1.40)	0.794	1.38 (1.02–1.87)	0.039
Dyslipidemia	0.94 (0.73–1.21)	0.625	0.99 (0.77–1.29)	0.990	1.01 (0.65–1.56)	0.967	1.05 (0.72–1.53)	0.789
Smoking	0.96 (0.78–1.18)	0.680	0.90 (0.73–1.12)	0.357	1.18 (0.80–1.73)	0.410	1.07 (0.77–1.49)	0.687

SMR: standardized mortality ratio; SVD: small vessel disease; WMH: white matter hyperintensities; CMB: cerebral microbleeds; CI: confidence interval; NAION: nonarteritic anterior ischemic optic neuropathy

### Adjusted data

After SMR weighting, the baseline characteristics were well-balanced between the 2 groups. The patients with NAION were more likely to have cerebral SVD. Among the details of cerebral SVD, only WMH was statistically significant, while CMB and silent lacunar infarcts were not (Table 1). The univariate logistic regression analysis showed that the OR of cerebral SVD was 3.73 times higher in patients with NAION than in the controls (95% CI, 1.78–7.80; p < 0.001). After adjusting for the effects of age, sex, hypertension, diabetes, dyslipidemia, and smoking by SMR, the OR of cerebral SVD was 4.86 times higher in patients with NAION than in the controls (95% CI, 2.10–11.24; p < 0.001) (Table 3). Among the subgroups of patients with cerebral SVD, only WMH showed a significant association with NAION, while CMB and silent lacunar infarcts did not.

**Table 3:** Multivariate logistic regression results for small vessel disease after SMR adjustment.

Variables	All cerebral SVD		WMH		CMB		Silent lacunar infarct	
	Odds ratio (95% CI)	p-value	Odds ratio (95% CI)	p-value	Odds ratio (95% CI)	p-value	Odds ratio (95% CI)	p-value
NAION	4.86 (2.10–11.24)	<0.001	4.44 (1.92–10.31)	0.001	2.26 (0.62–8.21)	0.217	2.92 (0.83–10.32)	0.096
Age (yr)	1.08 (1.03–1.13)	0.001	1.08 (1.03–1.13)	0.001	1.02 (0.96–1.08)	0.553	1.12 (1.05–1.20)	0.001
Sex (men)	1.19 (0.52–2.70)	0.684	0.88 (0.39–2.00)	0.757	1.29 (0.37–4.45)	0.691	1.43 (0.43–4.73)	0.560
Hypertension	1.40 (0.61–3.21)	0.431	1.37 (0.60–3.14)	0.458	1.15 (0.33–4.06)	0.830	4.75 (1.29–17.49)	0.019
Diabetes	1.63 (0.64–4.15)	0.306	2.00 (0.79–5.05)	0.144	0.57 (0.11–2.92)	0.502	0.36 (0.09–1.47)	0.155
Dyslipidemia	0.98 (0.33–2.89)	0.963	1.30 (0.44–3.85)	0.642	0.31 (0.03–3.73)	0.354	1.26 (0.28–5.74)	0.768
Smoking	0.91 (0.22–1.65)	0.323	0.62 (0.22–1.76)	0.369	0.95 (0.19–4.86)	0.951	1.83 (0.41–8.14)	0.427

SMR: standardized mortality ratio; SVD: small vessel disease; WMH: white matter hyperintensities; CMB: cerebral microbleeds; CI: confidence interval; NAION: nonarteritic anterior ischemic optic neuropathy

## Discussion

We evaluated the clinical information and results of brain MRIs in 63 patients with NAION and compared it to that of a control population with no neurological deficits. The NAION group tended to have a greater prevalence of vascular risk factors such as hypertension, diabetes mellitus, and dyslipidemia, as well as cerebral SVD. After adjusting for the effects of age and the vascular risk factors, the NAION group still had more cerebral SVD than the control group.

Among cerebrovascular diseases, there are three major etiologies: large artery atherosclerosis, SVD, and cardioembolism. Although the ultimate endpoints of all three subtypes include cerebral infarction and dementia, the causes, functional recoveries, and recurrence rates of each subtype are quite different.[17] Among them, cerebral SVD is more highly associated with chronic degenerative changes leading to functional and cognitive disabilities.[1] Conventionally, the mechanism of NAION has been debated–with the debates mainly based on whether the ischemia results from large artery atherosclerosis or thromboembolism from a remote source.[18–20] Hasanreisoglu et al.[21] reported that the risk of cardiovascular and cerebrovascular events following NAION is similar to that in the general population. On the other hand, in a prospective study of 406 patients with NAION, no increased risk of cerebrovascular disease or cardiac disease was reported.[5]

However, considering the mechanism of hypoperfusion in SPCA branches for NAION, and the similar target sites–arterioles–of both NAION and cerebral SVD, the connection between NAION and cerebral SVD can be postulated. Because complete evaluations of asymptomatic cerebral SVD require brain MRI with multiple protocols for WMH, CMB, and silent lacunar infarct, the relationship between cerebral SVD and NAION has not been easily investigated. In our study, even though there were some imbalances in baseline vascular risk factors between the NAION and control groups, the relationship between NAION and SVD was clearly observed, even after adjustment of this imbalance by both SMR-weighted analyses and conventional logistic regression analysis.

According to cerebral SVD, the three subsets (WMH, CMB, and silent lacunar infarcts) imply somewhat different meanings with respect to disease activity.[4] In the current study, the existence of WMH on brain MRI was robustly associated with NAION, while CMBs and silent lacunar infarcts were not statistically related to NAION. There are previous few studies that reported increased number of WMH in brain MRI of NAION patients.[22, 23] Although previous studies used age- and gender-matched controls, we approached by holistic weighting with age, sex and vascular risk factors using updated statistical methods. Additionally, they investigated the WMH findings from MRIs, not considering CMBs and silent lacunar infracts. Because cerebral SVD is composed of the three subsets (WMH, CMB, and silent lacunar infarcts), our study could be regarded as the first report to investigate the association between NAION and whole aspect of cerebral SVDs.

Among the three subsets of cerebral SVDs, WMHs exhibit the most chronic changes in cerebral small vessels via long-term hypoperfusion with incomplete infarction, not definite one. Silent lacunar infarcts are acute small vessel occlusions that lead to definite infarction at discrete timepoints without clinical symptoms due to their non-eloquent locations and small sizes.[24] CMB result from small ruptures of the proximal portions of cerebral small vessels without definite neurological events.[25] They share the same traits of fragility in cerebral small vessels with those of WMHs. However, acute features of CMBs are similar to those of silent lacunar infarcts. We found that patients with NAION were more likely to show the chronic markers of cerebral SVD (such as WMH) rather than the relatively acute characteristics of lacunar infarction. This suggests that patients with NAION share a similar pathophysiology with that of conditions associated with long-standing brain damage.

There are some limitations in our study. First, this was a retrospective study that included a small number of patients with NAION in one hospital compared to a much larger control group that may have a selection bias. Second, despite using well-adjusted controls with several variables, there may be additional missing confounds that could affect NAION and cerebral SVD. Third, because of distinct differences in the distribution of baseline characteristics between the two groups, we could not compare the prevalence of cerebral SVD directly. However, the powerful statistical techniques we utilized helped to reduce the effect of this discrepancy. Interestingly, smokers were significantly more frequent in the control group than NAION group in the present study. It may be that the controls, who are more concerned about their health status because of having risk factors such as smoking, are more likely to receive routine health checkup voluntarily.

Notwithstanding these limitations, the major strengths of this study are as follows: (1) the accuracy of the NAION diagnoses by the two neuro-ophthalmologists, (2) the large number of control participants with brain MRI scans, and (3) the reliability of the comparison between the two groups after adjusting age and vascular risk factors.

In conclusion, cerebral SVD was frequently present in patients with NAION compared with age and medical diagnosis-adjusted controls. Of interest, our study, which evaluated 63 patients with NAION and a control group, to the best of our knowledge, is the first to provide evidence demonstrating that NAION is associated with cerebral SVD. We suggest that patients with NAION should be considered for evaluation of cerebral SVD on brain MRI. Further prospective studies with large number of patients are warranted to elucidate the associations between SVD and NAION and to investigate the pathophysiology of NAION.
